# Causal effects of gut microbiome on hypertension: a Mendelian randomization study

**DOI:** 10.3389/fmicb.2023.1276050

**Published:** 2023-11-23

**Authors:** Gang He, Yu Cao, Houzhao Wang, Xiaoying Lv

**Affiliations:** ^1^Department of Blood Transfusion, Zhongshan Hospital (Xiamen), Fudan University, Xiamen, Fujian, China; ^2^School of Public Health, Xiamen University, Xiamen, Fujian, China; ^3^Department of Clinical Laboratory, Xiang‘an Hospital of Xiamen University, Xiamen, Fujian, China

**Keywords:** hypertension, gut microbiota, Mendelian randomization, causal relationship, blood pressure

## Abstract

**Background:**

Previous observational studies have shown that there is an important relationship between gut microbiota and hypertension, we performed a two-sample Mendelian randomization analysis to examine whether the gut microbiota is causally related to hypertension in order to find a basis for potential diagnostic or intervention approaches for hypertension.

**Methods:**

We obtained significant single nucleotide polymorphisms related to gut microbiota and hypertension from publicly available genome-wide association studies for a two-sample Mendelian randomization study. A total of 18,340 individual genome-wide genotype data were included from 24 population-based cohorts. The inverse-variance weighted meta-analysis is the main analytical method for evaluating causal relationships, and the Mendelian randomization research results have been validated through a series of sensitivity analyses.

**Results:**

The inverse-variance weighted analysis results indicated that phylum *Verrucomicrobia* (OR:0.831, 95%CI: 0.710–0.972; *p* = 0.021), family *BacteroidalesS24.7group* (OR:0.672, 95%CI: 0.496–0.911; *p* = 0.01), family *Bifidobacteriaceae* (OR:0.709, 95%CI:0.569–0.884, *p* = 0.002), genus *Adlercreutzia* (OR: 0.991, 95%CI: 0.982–0.999, *p* = 0.035), genus *Phascolarctacterium* (OR:0.819, 95%CI:0.685–0.981; *p* = 0.03), genus *LachnospiraceaeNK4A136group* (OR:0.990, 95%CI:0.981–0.999; *p* = 0.025), and genus *Ruminococcus2* (OR:0.988, 95%CI: 0.979–0.997; *p* = 0.008) had protective causal effects on hypertension. The Family *Alcaliginaceae* (OR:1.011, 95%CI:1.000–1.021, *p* = 0.04), Genus *Anaerostipes* (OR:1.375, 95%CI:1.096–1.653; *p* = 0.025), Genus *Collinsella* (OR:1.899, 95%CI:1.361–2.348; *p* = 0.02), and Genus *Lachnospiraceae_UCG_010* (OR:1.536, 95%CI:1.072–2.202; *p* = 0.019) were associated with a higher risk of HTN. The reverse Mendelian randomization analysis results showed no reverse causal relationship between HTN and these bacterial taxa.

**Conclusion:**

Our Mendelian randomization analysis results indicate a potential causal relationship between these bacterial taxa and hypertension, providing a new perspective for the treatment and prevention of hypertension.

## 1 Introduction

As a global public health concern, Hypertension (HTN) is related to a significant global burden of cardiovascular disease and premature death (Mills et al., 2020). Hypertension is also a leading hazard factor for cerebrovascular, cardiovascular, and chronic kidney diseases (Franklin and Wong, 2013; Mills et al., 2016; Kjeldsen, 2018). Globally, Compared to high-income countries, without effective intervention the increasing burden of hypertension in low- and middle-income countries will exacerbate the global epidemic of cardiovascular and kidney diseases. By 2010, more than 30% of the adult population (1.39 billion) suffered from hypertension, and hypertension is also recognized as the primary cause of global mortality (Zhou et al., 2021). The prevalence of hypertension is steadily rising worldwide due to factors such as an aging population and increased exposure to lifestyle risk factors, including unhealthy diets (such as high alcohol consumption, excessive sodium intake, and insufficient potassium intake) and lack of physical activity (Whelton, 2002; Louca et al., 2020). To effectively prevent and treat hypertension, it is crucial to gain a better understanding of the underlying mechanisms that contribute to its development. However, the exact cause of the increasing incidence rate of hypertension remains to be fully elucidated.

It is believed that the development process of hypertension is multifactorial, and one’s predisposition to hypertension is influenced by bot genetic and environmental factors, and the interaction of the two. A multitude of environmental factors increase the risk for HTN, including unbalanced diet, lack of physical activity, overweight and obesity, smoking, and psychological stress (Louca et al., 2020; Tsao et al., 2023). Recently, accumulating evidence has indicated that gut microbiota (GM) composition is closely related to human health and cardiovascular disease, including hypertension, which was strongly supported by at least three systematic reviews (Tang et al., 2017; Muralitharan et al., 2020; Louca et al., 2021). Considerable attention has been paid to the potential role of the gut microbiome in altering the development of hypertension, obesity, type-2 diabetes, and atherosclerosis (Tilg and Kaser, 2011; Howitt and Garrett, 2012; Karlsson et al., 2012; Qin et al., 2012; Tang et al., 2013; Yan et al., 2017). Studies have consistently shown that patients with hypertension exhibit dysbiosis in their gut microbiota, including reduced microbial richness, evenness, and diversity, and an increase in the *Firmicutes/Bacteroidetes* ratio (Yang et al., 2015).

Compared with germ-free mice that received an FMT (fecal microbiota transplantation) from 2 normotensive donors, germ-free mice that received FMT from a hypertensive human donor developed a significant increase in diastolic and systolic blood pressure after 8 weeks (Li et al., 2017; Muralitharan et al., 2020). In addition, daily consumption of probiotics for more than 8 weeks can significantly reduce diastolic and systolic blood pressure in hypertensive patients (Khalesi et al., 2014). Oral medication (minocycline) can also regulate blood pressure and normalize the ratio of *Firmicutes* to *Bacteroidetes* in spontaneously hypertensive rats and angiotensin II-induced hypertensive rats (Yang et al., 2015). The observational study showed that the composition and abundance of intestinal microbiota in HTN patients had significant changes compared with the healthy control group. However, while all this research evidence emphasizes the correlation between gut microbiota and HTN, it is still unclear which specific bacterial taxa lead to population differences (Yang et al., 2015; Yan et al., 2017). Confirming whether the correlation between gut microbiota and hypertension is causal and which microbiota taxa are the most important for hypertension is of great significance for the clinical practice of HTN management. Further research on the causal relationship between hypertension and gut microbiota will provide new prospects and perspectives for the treatment and prevention of hypertension and related diseases.

The traditional observational study is vulnerable to the influence of many potential factors such as lifestyle, socioeconomic status, and so on in the implementation process, which is prone to prejudice. Large randomized controlled trials (RCT) or cohort studies for a specific gut microbiome taxa are expensive however, so a new strategy is needed to study the causal effect of gut microbiome taxa on hypertension.

Mendelian randomization (MR) studies use genetic variations associated with modifiable exposure, typically single nucleotide polymorphisms (SNPs), to statistically evaluate the causal relationship between exposure and outcomes, in order to reduce confounding factors (lifestyle, socio-economic factors) and potential biases in reverse causality (Skrivankova et al., 2021). At the same time, MR research can overcome the shortcomings of extrapolation differences and data acquisition difficulties of traditional observational epidemiological research results. The purpose of this study is to explore the causal effects of gut microbiota on hypertension, systolic blood pressure (SBP), and diastolic blood pressure (DBP) using the Genome-Wide Association Study (GWAS) dataset through MR studies.

## 2 Materials and methods

The summary-level data used in this study was obtained from publicly available GWAS studies. Each cohort involved in the GWAS study received ethical approval and participation consent from their respective institutions, and aggregated data was published for analysis. In short, the gut microbiota is exposure, while hypertension is the outcome. This study employed stringent inclusion and exclusion criteria to select single nucleotide polymorphisms (SNPs) that are strongly associated with specific gut microbiota taxa as instrumental variables (IVs). Sensitivity analyses were performed to assess the robustness of the observed correlations. Furthermore, a reverse Mendelian randomization (MR) analysis was conducted to address potential confounding effects of hypertension on the causal relationship between gut microbiota and health outcomes.

In addition, the MR analysis relies on three key assumptions: (1) the instrumental variables used should exhibit a significant correlation with the exposure of interest. The strength of this correlation is typically evaluated using F-statistics, with a value of *F* ≥ 10 indicating no significant evidence of instrumental variable bias. If the F-statistic is less than 10, indicating a weak correlation, the corresponding instrumental variable is excluded. The formula for the F-statistic is F = (beta/se)^2. (2) The instrumental variables should be independent of confounding factors that may influence both the exposure and the outcomes. (3) There should be no horizontal pleiotropy, meaning that the instrumental variables only affect the outcomes through their impact on the exposure. Overall, the study employed rigorous methods to select instrumental variables and ensure the validity of the MR analysis.

### 2.1 Gut microbiota

The summary data of gut microbiota was obtained from a large-scale multi-ethnic GWAS coordinated by the MiBioGen consortium. As the largest human microbiome genetics study to date, a total of 18,340 individual genome-wide genotype data were included from 24 population-based cohorts (11 countries in Asia, Europe, North America, etc.) (Kurilshikov et al., 2021), and 22 cohorts are composed of adults or adolescents (*n* = 16,632), and two cohorts are composed of children (*n* = 1708). Among the 211 microbiome taxa, it includes five biological classifications: phylum, class, order, family, and genus. Five levels of IV of gut microbiome taxa were extracted from this large-scale GWAS to be applied in this study. The summary statistical data of the gut microbiota association research can be publicly available on the website www.mibiogen.org.

### 2.2 Hypertension

We obtained the outcome data (blood pressure) from the MR basic database, which is a well-planned database designed to ensure the effective implementation of the Mendelian randomization method. The MR-base database includes 1,674 GWAS datasets^1^ (Hemani et al., 2018). To identify relevant studies, we searched for keywords such as “hypertension,” “high blood pressure,” “systolic blood pressure,” and “diastolic blood pressure” in the MR-base database. We focused on studies conducted on the European population up to 2023.

Among the identified studies, we selected the one with the largest sample size as our outcome dataset. The selected dataset, with the ID “ukb-b-14177,” is from the MRC Integrative Epidemiology Unit (MRC-IEU) consortium based on the UK Biobank. The UK Biobank is a large and detailed prospective research institute that recruited over 500,000 participants aged 40 to 69 globally between 2006 and 2010 (Sudlow et al., 2015). The “ukb-b-14177” dataset includes 46,188 participants, with 2,076 cases and 460,857 controls. This dataset provides information on the diagnosis of hypertension by doctors. For the outcomes of systolic and diastolic blood pressure, we selected the datasets “ieu-b-38” and “ieu-b-39,” respectively. These datasets are based on the International Consortium for Blood Pressure (ICBP), which is a multi-stage design GWAS study on systolic and diastolic blood pressure for 200,000 Europeans (The International Consortium for Blood Pressure Genome-Wide Association Studies, 2011).^2^ The “ieu-b-38” and “ieu-b-39” datasets include summary-level data from the ICBP study (Evangelou et al., 2018) (Supplementary Tables). Please note that Supplementary Tables are available for further details.

### 2.3 Statistical analysis

All statistical analyses in this study were conducted using R software (version 4.1.2). We utilized the R software package “TwoSampleMR” to perform MR analysis investigating the causal relationship between the GM classification group and hypertension. The evaluation indicators for assessing the magnitude of each specific microbiota effect in MR studies were odds ratio (OR) and 95% confidence interval (95% CI). A statistical significance level of *p* < 0.05 was considered as evidence of potential causal effects (Waters and Ley, 2019; Xiang et al., 2021).

To ensure the authenticity and accuracy of the causal relationship between gut microbiome and hypertension, we implemented quality control measures to eliminate the interference of strong linkage imbalance caused by SNPs. This was accomplished through a series of screening settings: (1) SNPs with a value of *p* threshold of 1 × 10–5 were identified based on the genetic group of 18,000 European individuals; (2) The clumping distance between two SNPs was set to 10,000 kb; (3) The correlation coefficient r2 threshold of linkage disequilibrium (LD) between genes is set to 0.001. (4) Palindrome SNPs were removed to prevent the influence of alleles on the causal relationship between gut microbiome taxa and hypertension. (5) In cases where there was no SNP associated with exposure in the outcome GWAS, a proxy SNP significantly associated with the variation of interest (*r*^2^ > 0.8) was selected.

The primary analysis method used in this Mendelian randomization (MR) study was inverse variance weighted (IVW) (Burgess et al., 2013; Wang et al., 2021). IVW is a meta-analysis technique that combines ratio estimates with inverse variance weighting, ensuring the validity of each instrumental variable (IV) and accounting for SNP heterogeneity (Burgess et al., 2013; Bowden et al., 2017; Liu et al., 2022). The MR-Egger method, on the other hand, includes an intercept term in the weighted regression to assess horizontal pleiotropy among IVs (Burgess et al., 2017). The presence of a non-zero intercept suggests the presence of horizontal pleiotropy. While MR-Egger provides an estimate of the causal effect, it is less statistically efficient (Bowden et al., 2015). In contrast, the weighted median approach is able to provide consistent estimates of causal effects even when more than 50% of IVs are invalid (Hartwig et al., 2017). The weighted median method has advantages over MR-Egger in terms of result accuracy and maintaining a more precise causal effect estimate (Bowden et al., 2016; Xiang et al., 2021). Additionally, weighted mode and simple mode were used as additional methods for MR analysis (Hartwig et al., 2017; Wu et al., 2020).

To ensure the reliability and robustness of the causality assessment results, sensitivity analyses were performed. Cochrane’s *Q*-test was used to assess heterogeneity among the selected SNPs associated with each bacterial taxa. A value of *p* < 0.05 indicated significant heterogeneity among the IVs. MR-Egger regression was used to test for horizontal pleiotropy among the included SNPs. Furthermore, a weighted median analysis was conducted, which is more robust to individual genetic variants with strong outlier causality estimates. To investigate the causal effect of hypertension (HTN) on the identified significant bacterial genus, a reverse MR analysis was performed (i.e., HTN as exposure and the identified causal bacterial genus as outcome) using SNPs associated with HTN as IVs.

## 3 Results

Table 1 shows the results of pleiotropy and heterogeneity tests for all bacterial taxa (phylum, order, family, genus) included in the study. In sensitivity analysis, we confirmed the impact of accurate MR results from one phylum, one order, three families, and seven genera on HTN.

**Table 1:** The results of pleiotropy and heterogeneity tests for all bacterial taxa.

Outcome	Exposure	Heterogeneity				Pleiotropy		MR-PRESSO	
		MR Egger		IVW		MR Egger		Global Test	
		Cochran’s *Q*	*P*-value	Cochran’s *Q*	*P*-value	Egger intercept	*P*-value	RSSobs	*P*-value
Systolic blood pressure	Family *BacteroidalesS24.7* group	6.417410448	0.267694319	6.589411516	0.360490811	−0.025533642	0.729287544	8.980260819	0.398
	Family *Prevotellaceae*	23.4754342	0.036312445	23.5033307	0.052556821	0.005568384	0.902986166	26.83959231	0.062
	Genus *Eubacteriumbrachy* group	20.2397522	0.00250993	20.25789365	0.005038763	0.008216542	0.943923509	26.52558077	0.02
	Genus *Collinsella*	4.715105303	0.580837017	5.02017508	0.657501049	0.027865662	0.600675254	6.568510054	0.662
	Genus *Lachnospiraceae UCG010*	6.342391837	0.385947644	8.085511126	0.325114084	−0.047622043	0.246446396	12.40471335	0.288
	Genus *Prevotella7*	29.91594433	0.000218733	30.02320844	0.00043475	0.023547219	0.869714619	37.10243428	<0.002
	Genus *Subdoligranulum*	20.54281123	0.014842612	22.04661013	0.014868904	−0.039179277	0.4379134	26.46365238	0.016
	Genus *Terrisporobacter*	12.78759604	0.005119199	12.97465967	0.011400277	0.020091871	0.847486003	18.86236513	0.104
Diastolic blood pressure	Family *Bifidobacteriaceae*	15.02526844	0.09024423	15.11111161	0.128063133	−0.006208448	0.825678433	17.6722745	0.178
	Family *Prevotellaceae*	17.95935564	0.159068812	17.97445667	0.207946692	0.002359752	0.918327686	20.44074244	0.264
	Genus *Anaerostipes*	13.47582365	0.142230941	14.19156678	0.164431622	−0.018258691	0.506762232	16.92058235	0.204
	Genus *Escherichia.Shigell*a	27.10840464	0.000318633	27.80392816	0.000513003	0.020538919	0.684435849	35.0228526	<0.002
	Genus *Phascolarctobacterium*	6.660894863	0.465019933	7.145382935	0.52103139	0.025052863	0.508840225	8.975238731	0.554
	Genus *Prevotella7*	46.40211668	1.99315E-07	46.40628768	5.06148E-07	−0.002663586	0.979263265	57.00583207	<0.002
	Genus *Terrisporobacter*	3.952220093	0.266669933	6.167996805	0.186948539	0.039684959	0.285404614	11.23244775	0.26
	Order *Bifidobacteriales*	15.02526844	0.09024423	15.11111161	0.128063133	−0.006208448	0.825678433	17.6722745	0.184
	Phylum *Verrucomicrobia*	10.8592147	0.368591242	11.07836421	0.436721724	0.009009972	0.662843233	13.07632936	0.49
Vascular/heart problems	Family *Alcaligenaceae*	8.984902097	0.533537023	9.632878335	0.563680602	−0.001199876	0.439556577	11.47265556	0.578
diagnosed by doctor:	Genus *Eubacteriumbrachy* group	7.982163269	0.435214413	11.08297392	0.270064365	0.00238492	0.116285204	13.57264127	0.32
High blood pressure	Genus *Adlercreutzia*	5.827899736	0.442742582	6.204478903	0.516086812	0.001102424	0.561967397	8.155085342	0.514
	Genus *Lachnospiraceae* NK4A136 group	17.81451668	0.164689992	19.12433776	0.160252445	−0.00068179	0.346083125	21.84675528	0.184
	Genus *Ruminococcus2*	17.04735469	0.197148326	17.12865265	0.24938676	0.000241436	0.807258568	19.56599246	0.254
	Genus *Terrisporobacter*	4.165436759	0.244145183	4.613481409	0.329302769	0.000927873	0.609733334	6.918175552	0.424
	Order *Burkholderiales*	17.01729425	0.048445838	18.49378295	0.047183938	−0.001440391	0.399862741	22.28472063	0.058

**Figure 1:**
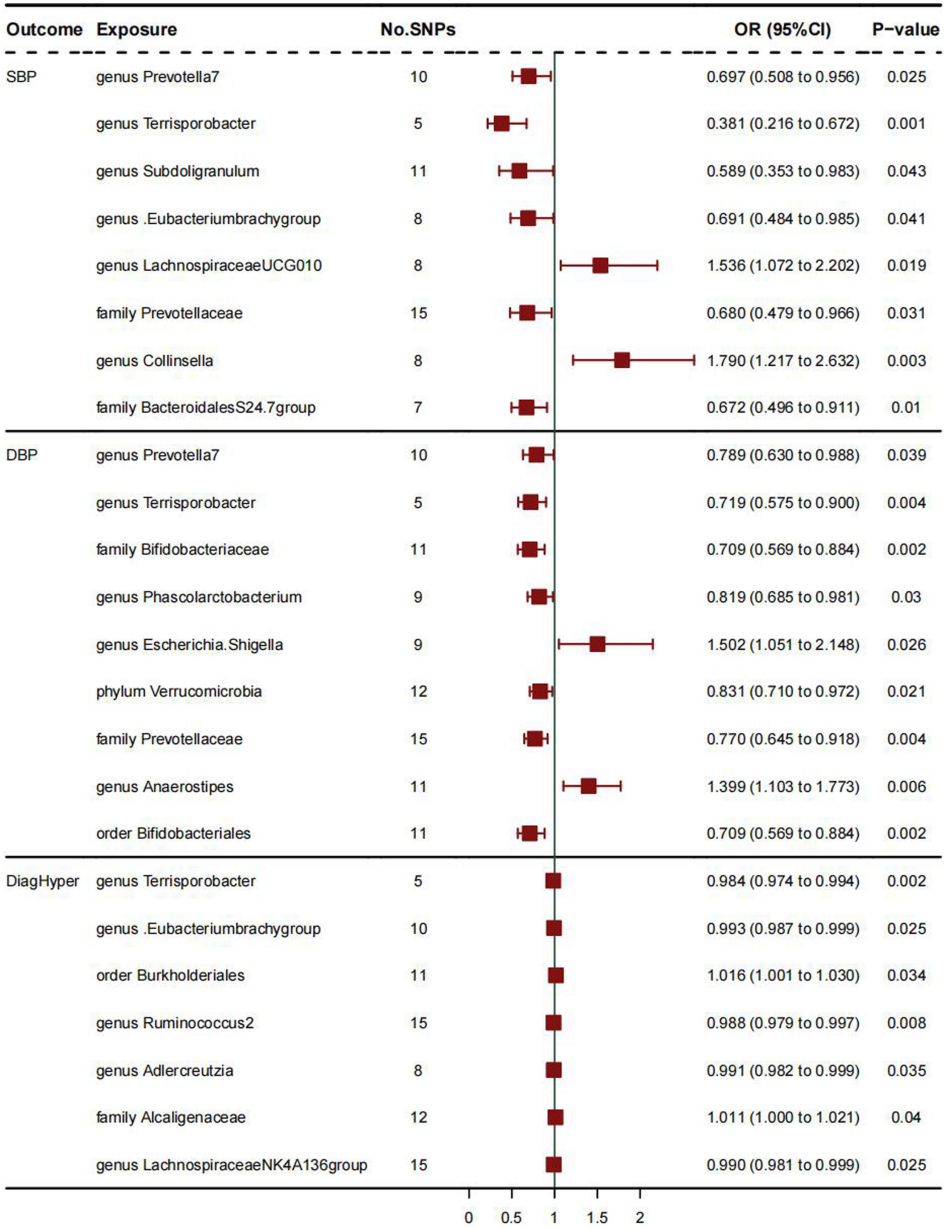
The results of Mendelian randomization analysis of three subtypes of hypertension and gut microbiota.

Supplementary Tables show the relationship between 211 gut microbiome taxa and HTN; Figure 1 shows the results of Mendelian randomization analysis of three subtypes of hypertension and gut microbiota. For SBP, the IVW results showed a positive correlation between two bacterial traits: Genus *Collinsella* (OR:1.790, 95%CI:1.217–2.632; *p* = 0.003), Genus *Lachnospiraceae_UCG_010* (OR:1.536, 95%CI: 1.072–2.202; *p* = 0.019) and systolic blood pressure, indicating that these two bacteria are risk factors for hypertension. And family *BacteroidalesS24.7group* (OR:0.672, 95%CI:0.496–0.911; *p* = 0.01) is negatively correlated with systolic blood pressure, indicating that these bacterial traits are protective factors for hypertension. The weighted median MR estimation also indicates that genus *Collinsella* (OR:1.899, 95%CI:1.361–2.348; *p* = 0.02) is a risk factor for systolic blood pressure.

As for DBP, IVW analysis results showed a positive correlation between genus *Anaerostipes* (OR:1.399, 95%CI:1.103–1.773; *p* = 0.006) and diastolic blood pressure, and these bacterial traits are a risk factor for diastolic blood pressure. The weighted mode MR analysis results of Genus *Anaerostipes* (OR:1.375, 95%CI:1.096–1.653; *p* = 0.025) also support this viewpoint. The IVW analysis results of bacterial traits: family *Bifidobacteriaceae* (OR:0.709, 95%CI:0.569–0.884, *p* = 0.002), genus *Phascolarctobacterium* (OR:0.819, 95%CI:0.685–0.981; *p* = 0.03), order *Bifidobacte- rials* (OR:0.709, 95%CI:0.569–0.884; p = 0.002), phylum *Verrucomimicrobia* (OR:0.831, 95%CI:0.710–0.972; *p* = 0.021) indicate that they are protective factors for diastolic blood pressure and negatively correlated with diastolic blood pressure. The MR analysis results of other weighted median values indicate that order *Bifidobacterials* (OR:0.738, 95%CI: 0.478–0.998; *p* = 0.022) is negatively correlated with diastolic blood pressure and has a protective effect on it.

As for DiagHyper (vascular/cardiac problem diagnosed by doctors: hypertension), IVW analysis results showed that family *Alcaliginaceae* (OR:1.011, 95%CI: 1.000–1.021, *p* = 0.04) was positively correlated with hypertension, indicating that these two bacteria are risk factors for hypertension, while genus *Adlercreutzia* (OR: 0.991, 95%CI:0.982–0.999, *p* = 0.035), genus *Lachnospiraceae NK4A136 group* (OR:0.990, 95%CI:0.981–0.999; *p* = 0.025), genus *Ruminococcus2* (OR:0.988, 95%CI:0.979–0.997; *p* = 0.008) were negatively correlated with hypertension, indicating that these bacteria traits are protective factors for hypertension.

## 4 Discussion

This Two-sample MR study is the first to analyze the causal relationship between gut microbiome taxa and hypertension through multiple datasets. After sensitivity analysis and reverse causality analysis, and the deletion of gut microbiota taxa lacking validity and reliability, The research results indicate that the levels of phylum *Verrucomimicrobia*, family *BacteroidalesS24.7group*, family *Bifidobacteriaceae*, genus *Adlercreutzia*, genus *Phascolarctacterium*, genus *Lachnospiraceae NK4A136 group*, and genus *Ruminococcus2* are negatively correlated with the risk of hypertension, and have a protective causal effect on the pathogenesis of HTN. Family *Alcaliginaceae*, Genus *Anaerostipes*, Genus *Collinsella*, and Genus *Lachnospiraceae- _UCG_010* may be risk factors for the onset of hypertension. The results were examined through some sensitivity analyses—MR-Egger analysis, IVW analysis, and MR-PRESSO Global Test analysis (Verbanck et al., 2018), which is consistent with our findings, and may promote the study of novel biomarkers in future HTN experiments. In the meantime, our results provide novel insights for future HTN prevention and therapeutic treatments: targeted regulation of dysbiosis of specific gut microbiome taxa to prevent and treat HTN.

The gut microbiota has the characteristic of diversity, it is mainly made up of 4 phyla: (1) *Firmicutes*, (2) *Bacteroidetes*, (3) *Actinobacteria*, and (4) *Proteobacteria*. The relative balance of gut microbiota composition plays a key role in maintaining intestinal immunity and systemic homeostasis; the imbalance of gut microbiota is often referred to as microecological imbalance, which is marked by the ratio of *Firmicutes* (F) to *Bacteroides* (B), compared to changes in the microbiota of healthy individuals (Guarner and Malagelada, 2003). Furthermore, some bacteria from the phylum *Firmicutes* are important producers of metabolic products that lower blood pressure, such as short-chain fatty acids (Petersen and Round, 2014). A multitude of studies indicated that there is an association between gut microbiota and hypertension (Yang et al., 2015; Li et al., 2017; Sun et al., 2019). The effect of gut microbiota on blood pressure regulation may be partially explained by the production of short-chain fatty acids (SCFAs) by gut bacteria, including beneficial SCFAs (acetate, butyrate, and propionate) and non-beneficial lactates. Meanwhile, Beli et al. suggested that gut microbiota interventions would be a new method for the prevention and treatment of HTN (Jose and Raj, 2015).

Considering the GM classification group at the phylum level, we found that Phylum *Verrucomimicrobia* is a protective factor for diastolic blood pressure. *Verrucomimicrobia* exists in the inner layer of the intestinal mucosa and is abundant in healthy individuals. They can decompose polysaccharides such as mucopolysaccharides and cellulose, providing energy and nutrients. The *Verrucomimicrobia* can also produce short-chain fatty acids, such as propionic acid and butyric acid, which play an important role in regulating intestinal health and the immune system (Schlesner et al., 2006). At the class level, we did not find a causal relationship between the GM taxa and HTN. It may be because refining the interactions between different taxonomic groups (such as the level of families and genera) can affect the observation results.

Furthermore, at the order level, we found that order *Bifidobacteriales* has a protective causal effect on diastolic blood pressure. Studies have shown that the abundance of *bifidobacteria* is higher in the healthy control group than in HTN patients (Peng et al., 2018). Short-chain fatty acids are produced in the process of fiber fermentation that are difficult to digest, and are one of the most characteristic microbial-derived metabolites. Acetate, propionate, and butyrate are three SCFAs with high abundance (Verhaar et al., 2020). The abundance of *bifidobacteria* in hypertension patients is lower, while *Bifidobacterium*, *Enterococcus,* and *Lactobacillus* are considered as probiotics. These three SCFA-producing microbes can produce SCFA and have multiple health benefits such as anti-inflammatory and beneficial metabolic effects (Hiippala et al., 2018; Parada Venegas et al., 2019). In addition, oral treatment of gut microbiota (specific *bifidobacteria*, *lactobacilli*, and SCFA-producing *Anaerobutyricum soehngenii* species that produce short-chain fatty acids) has a moderate antihypertensive effect on humans (Khalesi et al., 2014; Gilijamse et al., 2020).

At the family level, the family *BacteroidalesS24.7group* is a protective factor for systolic and diastolic blood pressure; research has shown a positive correlation between *Bacteroides* and blood pressure (Palmu et al., 2020). The family *Bifidobacteriaceae* belongs to the order *Bifidobacteriales*, and the analysis of the family *Bifidobacteriaceae* is as above. Family *Alcaliginaceae* is a risk factor for hypertension. Family *Alcaliginaceae*: In animal experiments, it was observed that after FMT transplantation in spontaneously hypertensive rats with normal blood pressure rats, the abundance of family *Alcaliginaceae* decreased in the gut (Adnan et al., 2017).

Unlike other GM and hypertension studies, we further identified three taxa at the genus level that increased the systolic blood pressure risk, five taxa at the genus level that increased the diastolic blood pressure risk, and four taxa at the genus level that increased the hypertension risk. The research results also showed that the genus *adlercreutzia* is negatively associated with BP Indices (Dan et al., 2019). Palmu et al. (2020) found that Genu *Collinsella* is Positively Associated with BP Indices. Meanwhile, cross-sectional studies in humans showed that the gut microbiota of symptomatic atherosclerosis patients had a higher abundance of the *Collinsella* genus, *Enterobacteriaceae*, *Streptococcaceae*, and *Klebsiella* spp., and a lower abundance of bacteria *Eubacterium*, *Roseburia*, and *Ruminococcaceae* spp. that can produce short chain fatty acids compared with the healthy control group (Karlsson et al., 2012; Jie et al., 2017; Liu et al., 2019). In animal models of hypertension complications (acute myocardial infarction), the gut microbiome, especially the *Lachnospiraceae* family, *Syntrophomonadaceae* family, and *Tissierella soehngenia* genus, exhibit a higher trend (Wu et al., 2017). Cross-sectional studies on gut microbiota composition in hypertension in humans showed a lower abundance of genus *Anaerostipes* in HT. Salt intake in diets will affect the incidence rate of hypertension and the composition of intestinal microbiota. In animal trials, higher salt intake is associated with changes in microbial community composition, including an increase in *Ruminococcus* and *Lachnospiraceae*, as well as a decrease in *Lactobacillus* and *Oscillibacter* (Wilck et al., 2017; Bier et al., 2018). Butyrate is a kind of SCFA, and the microbial community that produces butyric acid includes bacteria from families *Ruminococcaceae* and *Lachnospiraceae*, as well as *Anaerobutyricum hallii* and *Anaerostipes* spp. Our research results also indicated a negative correlation between the *Lactobacillus* genus and some *Lachnospiraceae* genera and blood pressure. The reduction of butyric-acid-producing bacteria is related to inflammatory diseases (including diabetes, obesity, hypertension, and inflammatory bowel disease), which is because butyric acid has an anti-inflammatory effect (Bach Knudsen et al., 2018; Li et al., 2018). Previous studies have shown that butyrate, as the main energy source of colon cells, can regulate tight junction proteins and maintain intestinal barrier integrity (Wu et al., 2019). Onyszkiewicz et al. (2019) found that butyrate enters the bloodstream after passing through the intestinal vascular barrier, and has a vasodilation effect on the mesenteric artery. This process occurs after acting on the G-protein coupled receptor (GPR) (Onyszkiewicz et al., 2019). Wang et al. (2017) found that sodium-butyrate can also inhibit ANGII induced hypertension by inhibiting the renin-angiotensin system mediated by the renin-(pro) renin receptor. In short, butyric acid may play an important role as a differentially beneficial metabolite in the regulation of hypertension. When analyzing the microbiota composition, a negative correlation between the SCFA-producing taxa *Clostridiaceae*, *Ruminococcus*, and *Coprococcus* on women and systolic BP was observed (Durgan, 2017). Bacteria within the *Ruminococcaceae* family are the key SCFAs-producing bacteria, which also play a crucial role in maintaining homeostasis and gut development (Biddle et al., 2013).

The main advantage of our study is that it is the first to use MR analysis to examine the relationship between gut microbiota and hypertension. This approach reduces confounding factors and provides more reliable results compared to observational studies. Additionally, our findings highlight the potential role of *Verrucomimicrobia* in the development of hypertension, which has not been previously reported. This suggests that *Verrucomicronia* may serve as a new biomarker for hypertension. However, our study does have some limitations. First, in terms of sample size, the gut microbiota GWAS contains a relatively small number of samples. Second, the MR study cannot determine whether there is data overlap in the included GWAS summary data. Of course, we have minimized the bias of participant overlap using the F-statistic (*F* > 10). Third, in this MR analysis, we did not find a causal relationship with HTN at the class level. Researchers can expand the sample to explore the relationship between gut microbiome taxa and HTN at the class level in future research.

In summary, our study provides a comprehensive assessment of the causal relationship between gut microbiota and hypertension. We identified eight gut bacteria (phylum *Verrucomicrobia*, order *Bifidobacteriales*, family *BacteroidalesS24.7group*, family *Bifidobacteriaceae*, genus *Adlercreutzia*, genus *Phascolarctobacterium*, genus *Lachnospiraceae NK4A136 group*, and genus *Ruminococcus2*) that have a negative causal relationship with hypertension, making them potential protective factors. Additionally, we found four gut bacteria (family *Alcaligenaceae*, genus *Anaerostipes*, genus *Collinsella*, and genus *Lachnospiraceae_UCG_010*) that have a positive causal relationship with hypertension, indicating they are hazard factors. These strains may serve as new biomarkers for the treatment and prevention of hypertension, providing new insights into the mechanisms underlying gut microbiota-mediated hypertension.

## Data availability statement

The datasets presented in this study can be found in online repositories. The names of the repository/repositories and accession number(s) can be found in the article/Supplementary material.

## Ethics statement

Each cohort involved in the GWAS study received ethical approval and participation consent from their respective institutions, and aggregated data was published for analysis.

## Author contributions

GH: Conceptualization, Methodology, Writing – review & editing, Writing – original draft. YC: Conceptualization, Data curation, Investigation, Methodology, Software, Writing – original draft. HW: Supervision, Validation, Writing – review & editing. XL: Project administration, Resources, Supervision, Writing – review & editing.

## Footnotes

^1^https://gwas.Mrcieu.ac.uk/

^2^www.ncbi.nlm.nih.gov/projects/gap/gibin/student.cgistudy_id=phs00585.v1.p1
